# Supplementary material for: Immunoglobulin Superfamily Containing Leucine-Rich Repeat (Islr) Participates in IL-6-Mediated Crosstalk between Muscle and Brown Adipose Tissue to Regulate Energy Homeostasis
Source: Int J Mol Sci. 2022 Sep 2;23(17):10008. doi: 10.3390/ijms231710008 (PMC9455994; doi:10.3390/ijms231710008)
Supplement: Supplementary file 1 [file ijms-23-10008-s001.zip › ijms-1851970-supplementary.pdf]

# Islr Participates in IL-6-mediated Crosstalk between Muscle and BAT to Regulate Energy Homeostasis

Chang Liu, [1] Jin Liu, [1] Tongtong Wang, [1] Yang Su, [1] Lei Li, [1] Miaomiao Lan, [1] Yingying Yu, [1] Fan Liu, [1] Lei Xiong, [1] Kun Wang, [1] Meijing Chen, [1] Na Li, [1] Qing Xu, [1] Yue Hu, [1] Yuxin Jia, [1] Qingyong Meng[1*]

[1]State Key Laboratories for Agrobiotechnology, College of Biological Sciences, China Agricultural University, Yuanmingyuan West Road No. 2, Haidian District, Beijing 100193, China.

[*]Correspondence: qymeng@cau.edu.cn

Supplementary Information includes:

Supplementary Figures

Supplementary Tables

## Supplementary Figures

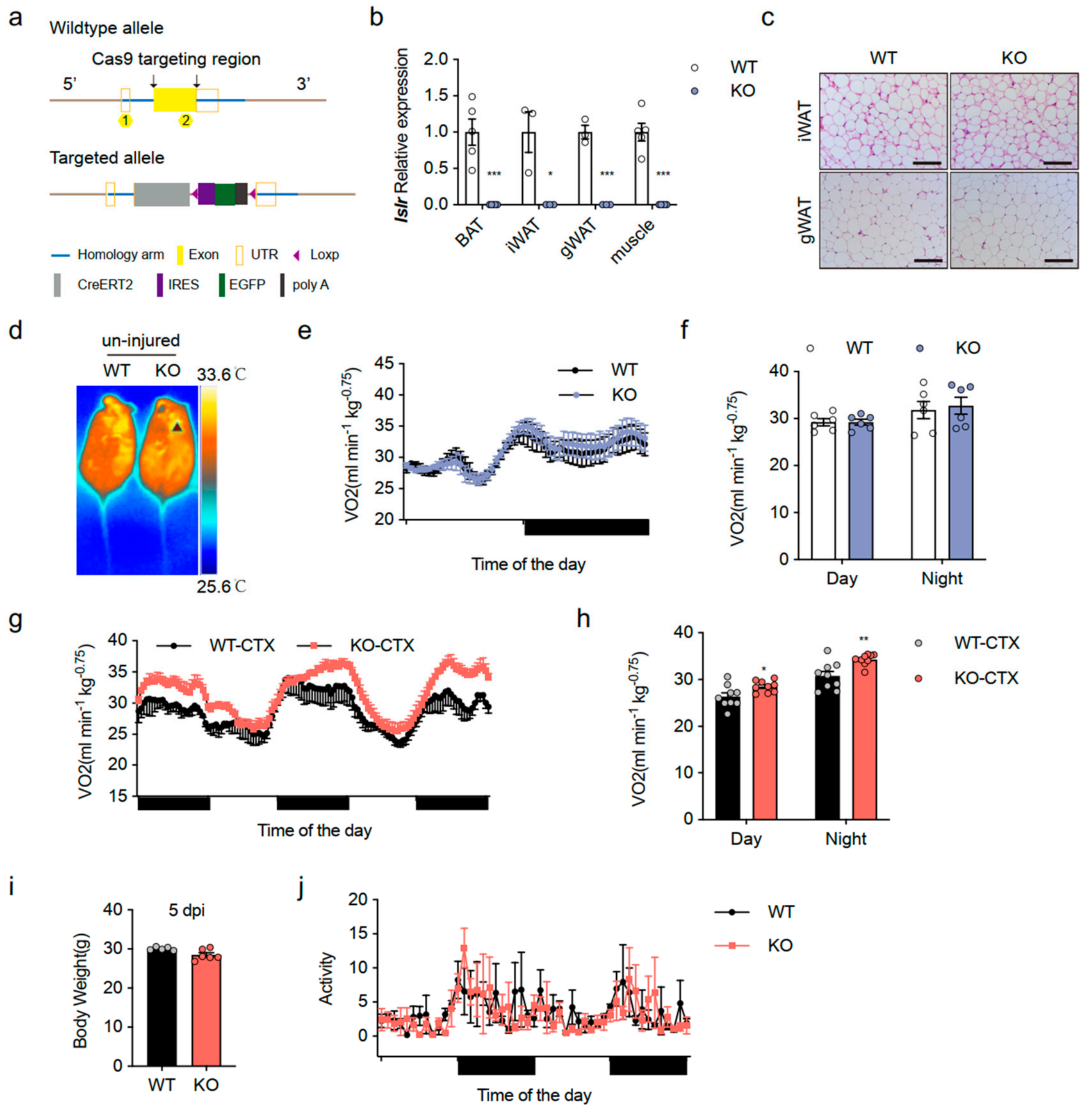


**Supplementary Figure S1 Islr deficiency elevates oxygen consumption upon muscle-BAT dialogue (relates to Figure 1)**

**a** Schematic diagram of the generation of KO mice.

**b** qRT-PCR analysis of Islr in multiple adipose tissues from WT and KO mice (n = 3-5) after CTX injury.

**c** Histological images of iWAT and gWAT from WT and KO mice at the age of 10 weeks after CTX injury (n = 3). Scale bar: 100 μm.

**d** Thermal images of the WT and KO mice under uninjured conditions.

**e**, **f** Indirect calorimetric analysis of $VO_2$ (**e**) and quantification of $VO_2$ (**f**) of WT and KO mice under uninjured conditions (n = 5-6).

**g, h** Indirect calorimetric analysis of $VO_2$ (**g**) and quantification of $VO_2$ (**h**) of WT and KO mice after CTX injury (n = 9).

**i**, **j** Body weight(**i**) and locomotor activity (**j**) of WT and KO mice following CTX injury (n = 5-6).

iWAT: inguinal WAT. gWAT: gonad WAT.

Error bars represent SEMs, $*P < 0.05$, $**P < 0.01$, and $***P < 0.001$, as determined by two-tailed Student's *t*-test.

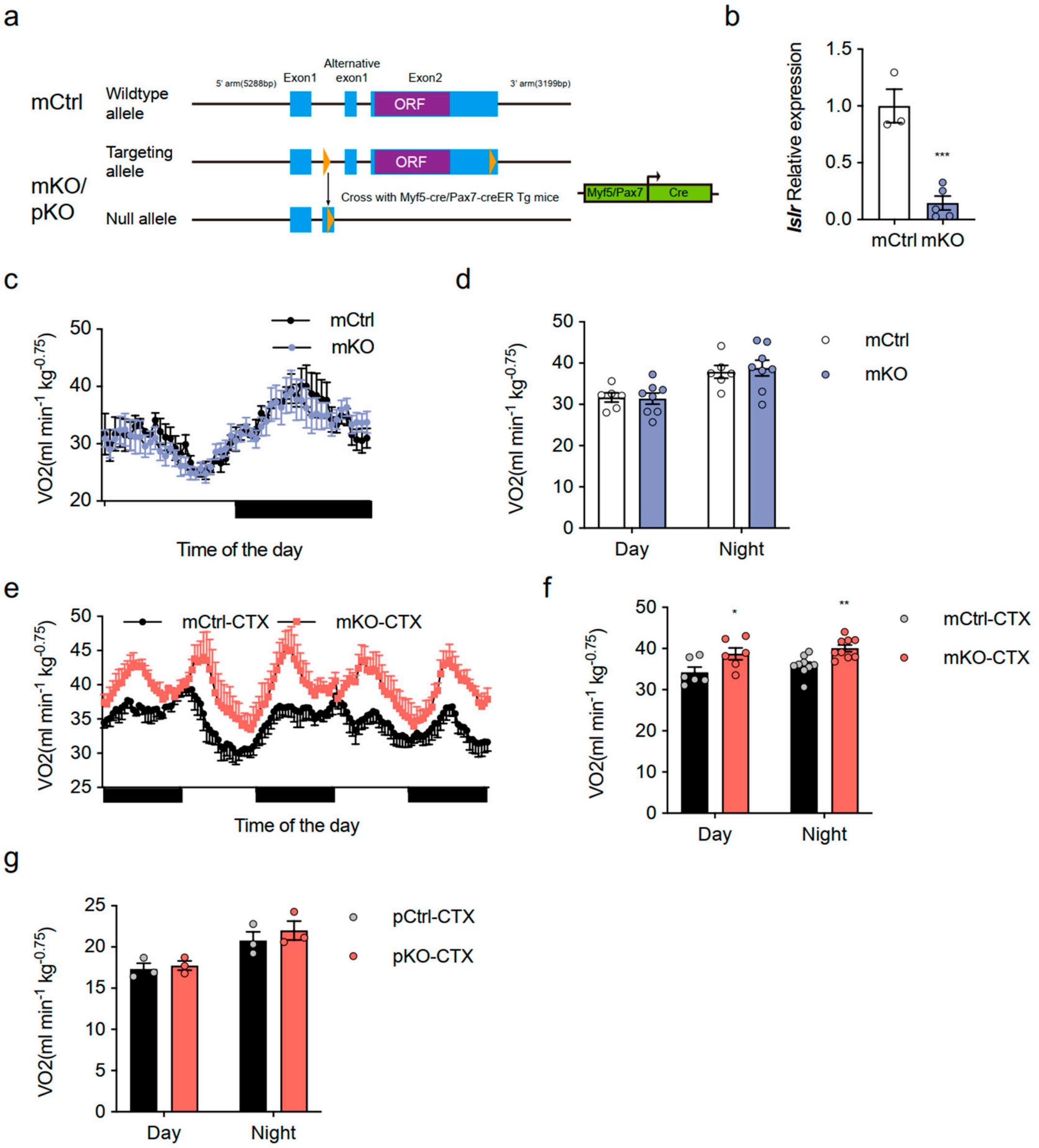


**Supplementary Figure S2. loss of Islr in BAT elevates oxygen consumption (relates to Figure 2)**

**a** Schematic diagram of the generation of mKO mice and pKO mice.

**b** qRT-PCR analysis of Islr in BAT from mCtrl and mKO mice (n = 3-5).

**c**, **d** Indirect calorimetric analysis of $VO_2$ (**c**) and quantification of $VO_2$ (**d**) of mCtrl and mKO mice under uninjured conditions (n = 6-8).

**e**, **f** Indirect calorimetric analysis of $VO_2$ (**e**) and quantification of $VO_2$ (**f**) of mCtrl and mKO mice after CTX injury (n = 6-9).

**g** Quantification of $VO_2$ of pCtrl and pKO mice after CTX injury (n = 3).

Error bars represent SEMs, *P < 0.05, **P < 0.01, and ***P < 0.001, as determined by two-tailed Student's *t*-test.

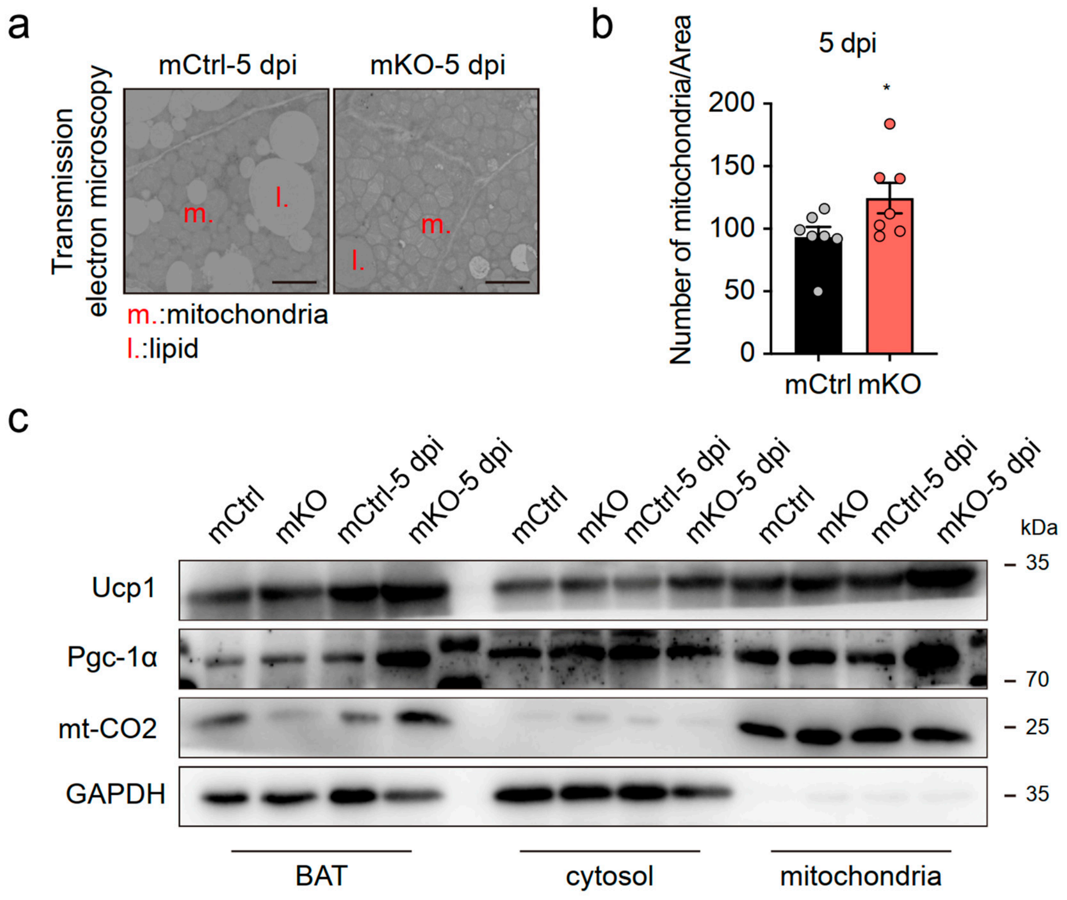


**Supplementary Figure S3. Loss of Islr induces mitochondrial activation in BAT (relates to Figure 3)**

a TEM images of BAT from mCtrl and mKO mice after CTX injury. Scale bar: 2.5 μm.

b Number of mitochondria per image of BAT from mCtrl and mKO mice (n = 7).

c Western blots for UCP1, PGC-1α and mt-CO2 in BAT, cytosol, and mitochondria of mCtrl and mKO mice after CTX injury. β-Tubulin was used as a loading control.

Error bars represent SEMs, $^*P < 0.05$, $^{**}P < 0.01$, and $^{***}P < 0.001$, as determined by two-tailed Student's *t*-test.

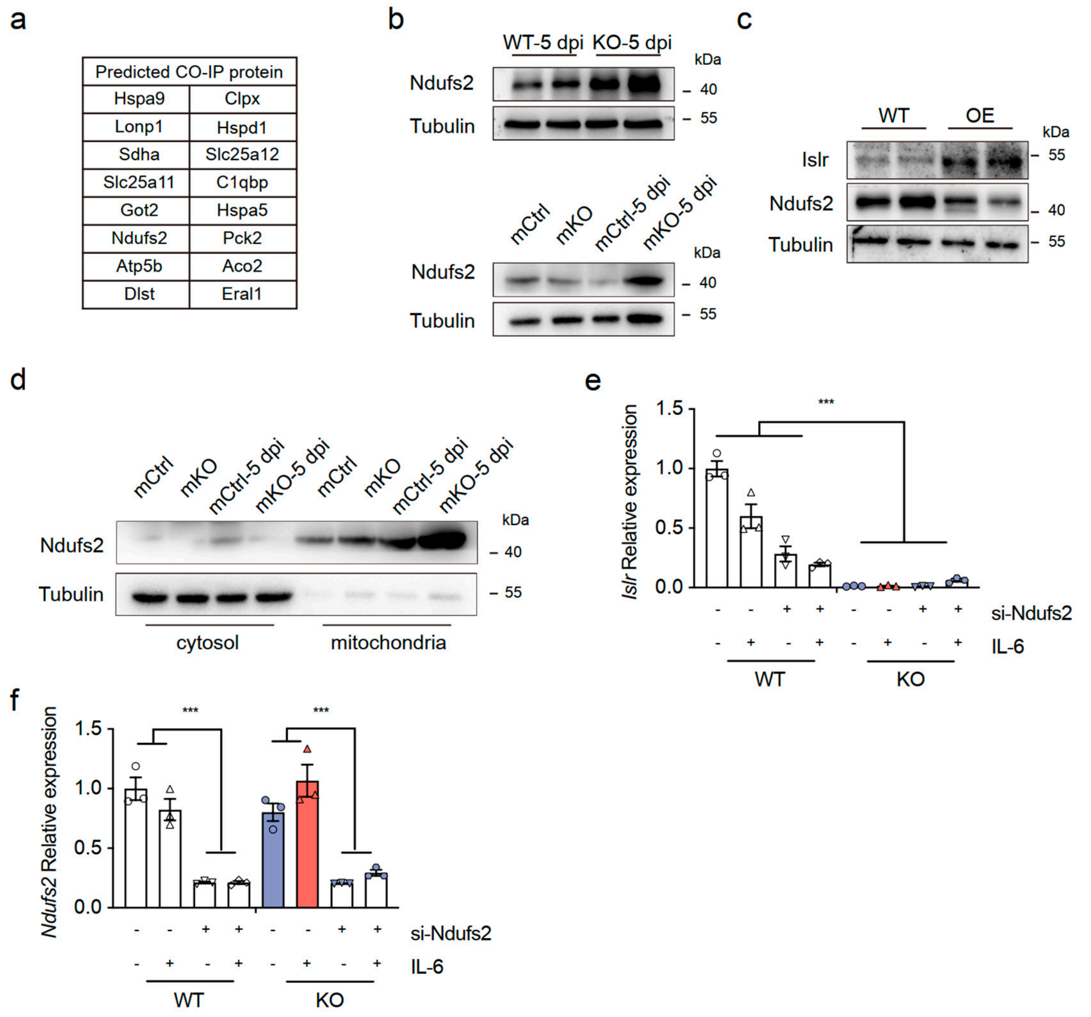


**Supplementary Figure S4. Islr is negatively correlated with Ndufs2 (relates to Figure 6)**

**a** The predict Islr interacted mitochondrial proteins of combined proteomics data with yeast two-hybrid database.

**b** Western blot analysis of Ndufs2 in BAT from WT and KO mice (mCtrl and mKO mice) after CTX injury. β-Tubulin was used as a loading control.

**c** Western blot analysis of Ndufs2 in BAT from WT and Islr-OE mice after CTX injury. β-Tubulin was used as a loading control.

**d** Western blot analysis of Ndufs2 in the cytosol and mitochondria of uninjured and CTX-injured mCtrl and mKO mice.

**e**, **f** qPCR analysis of Islr and Ndufs2 in differentiated BATSVF (day 6) upon Ndufs2 siRNA or IL-6 treatment ($n = 3$).

Error bars represent SEMs, *$P < 0.05$, **$P < 0.01$, and ***$P < 0.001$, as determined by two-tailed Student's *t*-test.

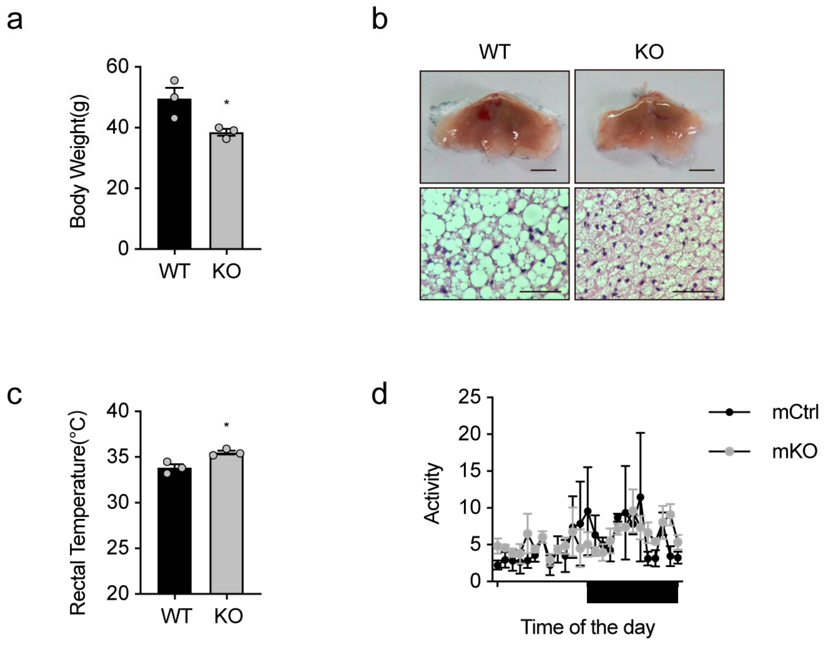


**Supplementary Figure S5 Loss of Islr prevents body weight gain by activating BAT in aging mice (relates to Figure 7)**

**a** Body weights of WT and KO mice at the age of 16 months (n = 3).

**b** Representative BAT and H&E staining of BAT sections from WT and KO mice at the age of 16 months. Scale bar: 0.5 cm and 50 μm.

**c** Quantification of the core body temperatures of WT and KO mice (n = 3).

**d** Locomotor activity of middle-aged mCtrl and mKO mice (n = 3).

Error bars represent SEMs, *P < 0.05, **P < 0.01, and ***P < 0.001, as determined by two-tailed Student's *t*-test.

## Supplementary Tables

### Supplementary Table S1. The antibodies used in the present study

| Antibody | Dilution | Catalog number | Company |
|---|---|---|---|
| Islr | 1:1000 | PAH781Hu01 | iCloud |
| GAPDH | 1:10000 | 5174S | CST |
| β-Tubulin | 1:10000 | ab6046 | Abcam |
| Islr | 1:1000 | HPA050811 | Sigma |
| Ndufs2 | 1:1000 | sc-390596 | Santa |
| PGC1α | 1:1000 | sc-518025 | Santa |
| UCP1 | 1:1000 | ab-10983 | Abcam |
| TFAM | 1:1000 | sc-166965 | Santa |
| Tom20 | 1:1000 | sc-17764 | Santa |
| mt-CO2 | 1:1000 | 55070-1-AP | Proteintech |
| OXPHOS | 1:1000 | ab-110413 | Abcam |

**Supplementary Table S2. Primers used in the present study**

| Genes | Forward | Reverse |
|---|---|---|
| *NRF1* | AGAAACGGAAACGGCCTCAT | CATCCAACGTGGCTCTGAGT |
| *NRF2* | ATGGAGCAAGTTTGGCAGGA | GCTGGGAACAGCGGTAGTAT |
| *DRP1* | ATGCCAGCAAGTCCACAGAA | TGTTCTCGGGCAGACAGTTT |
| *FIS1* | CAAAGAGGAACAGCGGGACT | ACAGCCCTCGCACATACTTT |
| *MFN1* | GCAGACAGCACATGGAGAGA | GATCCGATTCCGAGCTTCCG |
| *MFN2* | TGCACCGCCATATAGAGGAAG | TCTGCAGTGAACTGGCAATG |
| *OPA1* | ACCTTGCCAGTTTAGCTCCC | TTGGGACCTGCAGTGAAGAA |
| *Islr* | TGCGAGCAATCCAGTCCTTA | GGTTCAGGAGAACAGCCCAA |
| *Ucp1* | ACTGCCACACCTCCAGTCATT | CTTTGCCTCACTCAGGATTGG |
| *Pgc-1α* | AGCCGTGACCACTGACAACGAG | GCTGCATGGTTCTGAGTGCTAAG |
| *Tfam* | AAGGATGATTCGGCTCAGG | GGCTTTGAGACCTAACTGG |
| *mt-Nd4* | CTAATAATCGCACATGGCCTC | CGTAGTTGGAGTTTGCTAGG |
| *mt-Nd5* | CATCCTTCTCAACTTTACTGGG | TTTATGGGTGTAATGCGGT |
| *mt-Cyb* | CCATTCTACGCTCAATCCCCA | AGGCTTCGTTGCTTTGAGGTA |

**Table S2. Primers used in the present study**

| Genes | Forward | Reverse |
|---|---|---|
| *mt-Co1* | ACACAACTTTCTTTGATCCCG | AGAATCAGAACAGATGCTGG |
| *mt-Co2* | ATAATCCCAACAAACGACCT | CTCGGTTATCAACTTCTAGCA |
| *mt-Co3* | GGTATAATTCTATTCATCGTCTCGG | AGAACGCTCAGAAGAATCCT |
| *mt-Atp6* | CCTTCAATCCTATTCCCATCC | GTTGGAAAGAATGGAGACGG |
| *mt-Atp8* | GGCACCTTCACCAAAATCACT | GGGGTAATGAATGAGGCAAATAGA |
| *Ndufs2* | CAGCCAGATATTGAATGGGCA | TGTTGGTCACCGCTTTTTCCT |
| 36B4 | GCAGACAACGTGGGCTCCAAGCAGAT | GGTCCTCCTTGGTGAACACGAAGCCC |
